# Stimulation of MMP-1 and CCL2 by NAMPT in PDL Cells

**DOI:** 10.1155/2013/437123

**Published:** 2013-08-22

**Authors:** Marjan Nokhbehsaim, Sigrun Eick, Andressa Vilas Boas Nogueira, Per Hoffmann, Stefan Herms, Holger Fröhlich, Søren Jepsen, Andreas Jäger, Joni Augusto Cirelli, James Deschner

**Affiliations:** ^1^Experimental Dento-Maxillo-Facial Medicine, Center of Dento-Maxillo-Facial Medicine, University of Bonn, 53111 Bonn, Germany; ^2^Clinical Research Unit 208, Center of Dento-Maxillo-Facial Medicine, University of Bonn, Welschnonnenstraße 17, 53111 Bonn, Germany; ^3^Department of Periodontology, Laboratory of Oral Microbiology, University of Bern, 3010 Bern, Switzerland; ^4^Department of Diagnosis and Surgery, School of Dentistry, SP, UNESP, 14801-903 Araraquara, Brazil; ^5^Institute of Human Genetics, Biomedical Center, University of Bonn, 53127 Bonn, Germany; ^6^Division of Medical Genetics, University Hospital Basel and Department of Biomedicine, University of Basel, 4058 Basel, Switzerland; ^7^Bonn-Aachen International Center for IT, Algorithmic Bioinformatics, University of Bonn, 53113 Bonn, Germany; ^8^Department of Periodontology, Operative and Preventive Dentistry, University of Bonn, 53111 Bonn, Germany; ^9^Department of Orthodontics, University of Bonn, 53111 Bonn, Germany

## Abstract

Periodontitis is an inflammatory disease caused by pathogenic microorganisms and characterized by the destruction of the periodontium. Obese individuals have an increased risk of periodontitis, and elevated circulating levels of adipokines, such as nicotinamide phosphoribosyltransferase (NAMPT), may be a pathomechanistic link between both diseases. The aim of this in vitro study was to examine the regulation of periodontal ligament (PDL) cells by NAMPT and its production under inflammatory and infectious conditions. NAMPT caused a significant upregulation of 9 genes and downregulation of 3 genes, as analyzed by microarray analysis. Eight of these genes could be confirmed by real-time PCR: NAMPT induced a significant upregulation of EGR1, MMP-1, SYT7, ITPKA, CCL2, NTM, IGF2BP3, and NRP1. NAMPT also increased significantly the MMP-1 and CCL2 protein synthesis. NAMPT was significantly induced by interleukin-1**β** and the periodontal microorganism *P. gingivalis*. NAMPT may contribute to periodontitis through upregulation of MMP-1 and CCL2 in PDL cells. Increased NAMPT levels, as found in obesity, may therefore represent a mechanism whereby obesity could confer an increased risk of periodontitis. Furthermore, microbial and inflammatory signals may enhance the NAMPT synthesis in PDL cells and thereby contribute to the increased gingival and serum levels of this adipokine, as found in periodontitis.

## 1. Introduction

Periodontitis is a chronic inflammatory disease, which is characterized by the destruction of the tooth-supporting tissues, such as the periodontal ligament (PDL), and caused by pathogenic microorganisms, such as *Porphyromonas gingivalis*, *Tannerella forsythia*, *Treponema denticola*, and *Aggregatibacter actinomycetemcomitans*. These periodontopathogens can trigger local production of proinflammatory mediators, such as interleukin-1*β* (IL-1*β*), cyclooxygenase 2 (COX2), as well as chemokine, cc motif, ligand 2 (CCL2), and matrix-degrading proteases, such as matrix metalloproteinase-1 (MMP-1), by infiltrating and resident cells of the periodontium. As a consequence of the exaggerated immunoinflammatory and proteolytic processes, the periodontal tissues are subjected to degradation and resorption, which can finally result in tooth loss [1, 2]. Data from the National Health and Nutrition Examination Survey III, which analyzed the health and nutritional status in the United States, demonstrate that approximately half of the US population aged ≥30 years is afflicted with periodontitis [3]. Periodontitis is also associated with systemic diseases and conditions, such as cardiovascular diseases, diabetes mellitus, and obesity, and has a negative influence on a wide range of physical, psychological, and social aspects of quality of life [4–6]. 

 The exact mechanisms underlying the association between periodontitis and obesity are as yet unknown. However, it has been suggested that increased serum levels of adipokines derived from adipose tissue could make obese individuals more susceptible to periodontitis. The adipose tissue is not only an energy storage tissue but also acts as an endocrine organ, from which adipokines, such as nicotinamide phosphoribosyltransferase (NAMPT, also known as visfatin or pre-B-cell colony-enhancing factor 1), are secreted. Adipokines not only regulate insulin sensitivity and energy expenditure but also inflammatory and healing processes [7]. NAMPT is mainly produced by macrophages and adipocytes in the adipose tissue and induces production of inflammatory mediators and nuclear factor-kappaB (NF*κ*B) activation [8]. High serum levels of NAMPT are found in obesity, metabolic syndrome, type 2 diabetes, atherosclerosis, and other diseases [9–11]. Therefore, increased serum levels of NAMPT could be at least one mechanism, whereby these diseases contribute to periodontitis. Recently, it has been demonstrated that NAMPT is also present in high levels in gingival crevicular fluid (GCF), gingival tissues, and serum from periodontally diseased patients, indicating that NAMPT might also be produced locally in the periodontium and play a role in the etiopathogenesis of periodontitis [12, 13]. However, whether NAMPT regulates the gene expression and protein synthesis of periodontal cells has yet to be elucidated. Furthermore, whether NAMPT is induced in these cells by periodontopathogens and IL-1*β*, which has been shown to be increased in GCF and gingival tissues at inflamed sites, is also largely unknown. This study was therefore established to examine the actions of NAMPT and its regulation by microbial and inflammatory signals in human PDL cells. 

## 2. Materials and Methods

### 2.1. Culture and Treatment of Cells

PDL cells were harvested from periodontally healthy teeth that had to be extracted for orthodontic reasons. Written informed parental consent and approval of the Ethics Committee of the University of Bonn were obtained (#043/11). The cells were cultured in Dulbecco's minimal essential medium (DMEM, Invitrogen, Karlsruhe, Germany) supplemented with 10% fetal bovine serum (FBS, Invitrogen), 100 units penicillin, and 100 *μ*g/mL streptomycin (Biochrom, Berlin, Germany) at 37°C in a humidified atmosphere of 5% CO_2_. PDL cells from passages 3 to 5 were seeded (50,000 cells/well) on cell culture plates and grown to 80% confluence. One day prior to the experiment, the FBS concentration was reduced to 1%. Medium was changed every other day.

 In order to study the actions of NAMPT on PDL cells, various concentrations of NAMPT (30–300 ng/mL; Biomol, Hamburg, Germany) were added to cells. To mimic an inflammatory environment, cells were incubated with IL-1*β* (0.2–5 ng/mL; Calbiochem, San Diego, CA, USA), as done in our previous studies [14–16]. In order to simulate an infectious environment in vitro, cells were stimulated with the inactivated oral periodontopathogens *Porphyromonas gingivalis *ATCC 33277, *Tannerella forsythia *ATCC 43037, *Treponema denticola *ATCC 35405, and *Aggregatibacter actinomycetemcomitans* Y4 (optical density: 0.025, 0.05, and 0.1). Bacteria were suspended in PBS (OD_660 nm_ = 1, equivalent to 1.2 × 10^9^ bacterial cells/mL) and exposed two times to ultrasonication (160 W for 15 min) resulting in a complete killing. In the present study, cells were exposed to NAMPT, IL-1*β*, or periodontopathogens for up to 3 d. In order to unravel the intracellular mechanisms underlying the effects of NAMPT, PDL cells were preincubated with specific inhibitors against NF*κ*B (pyrrolidine dithiocarbamate, PDTC; 10 *μ*M; Calbiochem), JNK (SP600125; 10 *μ*M; Calbiochem), p38 (SB203580; 10 *μ*M; Calbiochem), MEK1/2 (U0126; 10 *μ*M; Calbiochem), or PI3 K (50 *μ*M; Calbiochem) signaling pathways 1 h before experiments.

### 2.2. Microarray Analysis

The regulatory effects of NAMPT on the gene expression of PDL cells were analyzed by a genomewide expression profiling using Illumina's HumanHT-12 v4 Expression BeadArrays (Illumina, San Diego, CA, USA). The RNA was reverse transcribed, amplified, and subsequently biotinylated using the Illumina TotalPrep-96 RNA Amplification Kit (Life Technologies, Carlsbad, CA, USA). The resulting cRNA was hybridized to the arrays according to the manufacturers manual using an automated liquid handling pipeline and scanned on an IScan System. The intensity signals were QC checked and exported using Illumina's GenomeStudio 2011 v1.1 software suite.

 All array data underwent a rigorous quality control, before applying background correction (subtraction of an offset, which is estimated from the control probes) and a subsequent variance stabilizing transformation [17], followed by quantile normalization [18]. All steps were conducted using the lumi R-package [19]. After calculation of present/absent calls we only kept probes, which were present in each patient in each group. In order to consider additional confounding factors, which might potentially influence gene expression but are not directly captured by the available patient information, we applied the algorithm by Leek and Storey [20], which estimates a surrogate variable model. In addition, relative array quality weights were computed via the limma R-package [21] using the algorithm by Ritchie et al. [22]. Both, surrogate variables and array weights, can be considered when fitting a linear model to each gene via limma. In our case, the limma model contained two additive factors, namely, a donor and a group factor; that is, essentially we were fitting 2-way ANOVA model. After model fitting, differential gene expression for all clinically relevant differences can be extracted as contrasts from the model and corresponding *P* values can be computed (multiple testing correction) [23]. We refer the reader to the limma user guide for an excellent introduction [24]. 

### 2.3. Real-Time PCR

RNA was extracted using an RNA extraction kit (Qiagen, Hilden, Germany), and a total of 1 *μ*g of RNA was reverse transcribed using iScriptTM Select cDNA Synthesis Kit (Bio-Rad Laboratories, Munich, Germany) at 42°C for 90 min followed by 85°C for 5 min. Expression of early growth response 1 (EGR1), MMP-1, synaptotagmin 7 (SYT7), inositol 1,4,5-trisphosphate 3-kinase a (ITPKA), CCL2, neurotrimin (NTM), insulin-like growth factor 2 mRNA-binding protein 3 (IGF2BP3), neuropilin 1 (NRP1), potassium channel tetramerization domain-containing protein 12 (KCTD12), heat-shock 27 kD protein 3 (HSPB3), transmembrane 4 L six family member 20 (TM4SF20), regulator of G protein signaling 4 (RGS4), NAMPT, COX2, and glyceraldehyde-3-phosphate dehydrogenase (GAPDH) was detected by real-time PCR using the iCycler iQ detection system (Bio-Rad Laboratories), SYBR Green (Bio-Rad Laboratories), and specific primers (QuantiTect Primer Assay, Qiagen). One *μ*L of cDNA was amplified as a template in a 25 *μ*L reaction mixture containing 12.5 *μ*L 2x QuantiFast SYBR Green PCR Master Mix (Qiagen), 2.5 *μ*L of primers, and deionized water. The mixture was heated initially at 95°C for 5 min and then followed by 40 cycles with denaturation at 95°C for 10 s and combined annealing/extension at 60°C for 30 s. GAPDH was used as an endogenous control. The data were analyzed by the comparative threshold cycle (CT) method. 

### 2.4. ELISA

The levels of NAMPT, MMP-1, and CCL2 in the supernatants of PDL cells were analysed by commercially available enzyme-linked immunoassay (ELISA) kits (RayBiotech, Norcross, GA, USA) according to the manufacturer's instructions. The absorbance was measured with a microplate reader (PowerWave x, BioTek Instruments, Winooski, VT, USA) at 450 nm. The data were normalized by the cell number, which was measured with an automatic cell counter (Moelab, Hilden, Germany).

### 2.5. Immunocytochemistry

PDL cells were cultured in the presence and absence of *P. gingivalis* ATCC 33277 or IL-1*β* on glass coverslips in 24-well plates. After 3 days, cells were fixed in 4% paraformaldehyde (Sigma-Aldrich, Munich, Germany) at pH 7.4 and room temperature (RT) for 15 min and then permeabilized in 0.1% Triton X-100 (Sigma-Aldrich) for 5 min. Non specific antigens were blocked by incubation with serum block (LSAB System; Santa Cruz Biotechnology, Santa Cruz, CA, USA) for 20 min. Subsequently, cells were incubated at RT for 90 min with rabbit polyclonal antibody to NAMPT (Santa Cruz Biotechnology). Afterwards cells were labeled with goat anti-rabbit IgG-HRP secondary antibody (Cell Signalling Technology, Danvers, MA, USA) for 45 min. For staining, cells were exposed to DAB chromogen (Dako, Hamburg, Germany) for 3 min at RT. After each incubation step, cells were washed twice with PBS (Sigma-Aldrich). Counterstaining was performed with Mayer's Haematoxylin (Merck Eurolab, Dietikon, Switzerland) for 2 min. Coverslips were mounted in DePex mounting medium (Serva Electrophoresis, Heidelberg, Germany). Standardized photomicrographs were taken using an Axioplan 2 imaging microscope (Carl Zeiss MicroImaging, Jena, Germany).

### 2.6. Immunofluorescence

PDL cells were fixed with 4% paraformaldehyde in PBS pH 7.4 for 10 min at RT, washed with PBS, and treated with 0.1% Triton X-100 for 5 min at RT. Then cells were washed again with PBS and blocked with a blocking buffer (nonfat dry milk; Bio-Rad Laboratories) for 1 h at RT. After washing, the cells were incubated with primary rabbit antibody NF*κ*B p65 (1 : 400; Cell Signalling Technology) for 90 min and with secondary antibody CY3 (1 : 2,000; Abcam, Cambridge, MA, USA) for 45 min. Cells were observed under a 20x objective using an Axioplan 2 imaging microscope (Carl Zeiss MicroImaging). The images were captured with a PVCAM camera and the VisiView capturing software (Visitron Systems, Puchheim, Germany).

### 2.7. Statistical Analysis

The IBM SPSS Statistics 20 software was used for analysis. Mean values and standard errors of the mean (SEM) were calculated. All experiments were performed in triplicate and repeated at least twice. For statistical analysis, parametric (*t* test and ANOVA followed by the post-hoc Dunnett's test) and non parametric tests (Wilcoxon and Mann-Whitney *U* tests) were applied. Differences between groups were considered significant at *P* < 0.05. Microarray data were analyzed in the Bonn-Aachen International Center for IT, Algorithmic Bioinformatics, University of Bonn, Germany (see Section 2.2).

## 3. Results

### 3.1. Regulation of Gene Expression by NAMPT in PDL Cells

**Figure 1:**
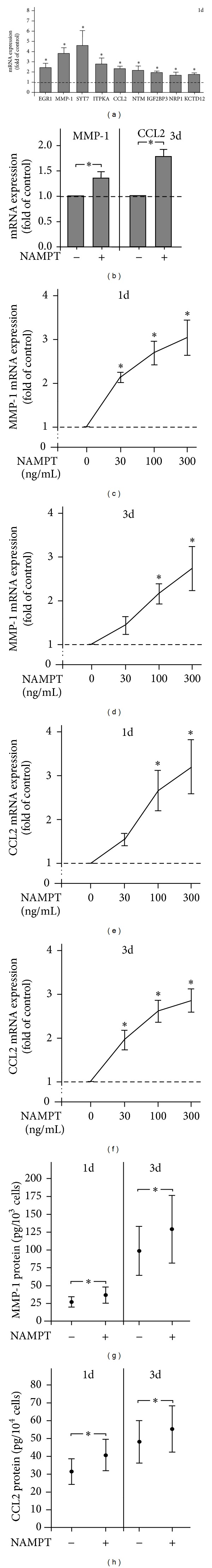
Stimulation of gene expression and protein synthesis by NAMPT. Upregulation of genes by NAMPT (100 ng/mL) in PDL cells from 10 donors at 1 d (a). Upregulation of MMP-1 and CCL2 expression by NAMPT (100 ng/mL) in PDL cells from 10 donors at 3 d (b). Stimulation of MMP-1 expression by various concentrations of NAMPT in PDL cells from 3 donors at 1 d (c) and 3 d (d). Stimulation of CCL2 expression by various concentrations of NAMPT in PDL cells from 3 donors at 1 d (e) and 3 d (f). Stimulation of MMP-1 protein synthesis by NAMPT (100 ng/mL) in PDL cells from 6 donors at 1 d and 3 d (g). Stimulation of CCL2 protein synthesis by NAMPT (100 ng/mL) in PDL cells from 6 donors at 1 d and 3 d (h). All experiments were performed in triplicate and repeated at least twice. Mean ± SEM; *significantly (*P* < 0.05) different from NAMPT-untreated cells (control).

First, we sought to clarify whether NAMPT regulates the expression of genes in PDL cells by performing a microarray-based approach. As shown in Table 1, NAMPT caused a significant upregulation of 9 genes and a significant downregulation of 3 genes in PDL cells from 3 donors at 1 d. The NAMPT-upregulated genes were the following: EGR1, MMP-1, SYT7, ITPKA, CCL2, NTM, IGF2BP3, NRP1, and KCTD12. The 3 NAMPT-downregulated genes comprised HSPB3, TM4SF20, and RGS4 (Table 1). In PDL cells from 10 donors, the results from the microarray analysis were then validated by real-time PCR, which confirmed 8 out of the 12 NAMPT-regulated genes: NAMPT induced a significant upregulation of the mRNA expression for EGR1 (2.4-fold), MMP-1 (3.8-fold), SYT7 (4.6-fold), ITPKA (2.8-fold), CCL2 (2.3-fold), NTM (2.1-fold), IGF2BP3 (1.9-fold), and NRP1 (1.7-fold). KCTD12 was also upregulated by NAMPT, but the effect did not reach statistical significance (Table 2 and Figure 1(a)). The effects of NAMPT on the remaining genes could not be confirmed by PCR (data not shown). 

### 3.2. Effect of NAMPT on MMP-1 and CCL2

Since MMP-1 and CCL2 are molecules known to be strongly associated with periodontitis, we further studied their regulation by NAMPT. As observed at 1 d, NAMPT increased significantly the MMP-1 (1.4-fold) and CCL2 (1.8-fold) expressions at 3 d (Figure 1(b)). Furthermore, the stimulatory effects of NAMPT on MMP-1 and CCL2 expressions were dose-dependent at both time points, as shown in Figures 1(c)–1(f). In addition, the NAMPT-induced upregulation of the MMP-1 and CCL2 expressions was parallelled by increased MMP-1 and CCL2 protein levels in the supernatants of NAMPT-stimulated cells, as compared to those of the control, at 1 d and 3 d (Figures 1(g) and 1(h)).

### 3.3. Exploitation of the JNK Pathway by NAMPT

**Figure 2:**
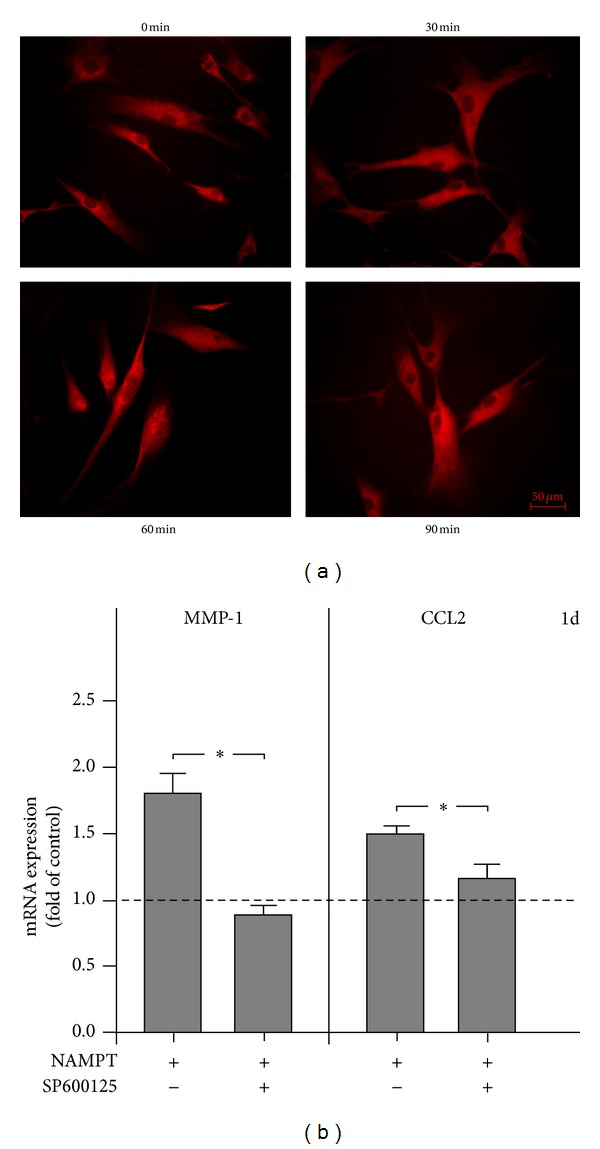
Intracellular pathways triggered by NAMPT. Stimulation of NF*κ*B nuclear translocation by NAMPT at 60 min (a). Inhibition of the NAMPT-stimulated MMP-1 and CCL2 upregulation by preincubation of PDL cells with SP600125, a selective inhibitor of JNK, at 1 d (b). Images and data from one representative donor are shown. All experiments were performed in triplicate and repeated at least twice. Mean ± SEM; *significant (*P* < 0.05) difference between groups.

Next, we studied how the effects of NAMPT on MMP-1 and CCL2 are mediated intracellularly. As evidenced by immunofluorescence microscopy, NAMPT stimulated the nuclear translocation of NF*κ*B and caused a maximal NF*κ*B accumulation within the nucleus at 60 min (Figure 2(a)). However, pre-incubation of cells with PDTC, a specific inhibitor of NF*κ*B, had not effect on the NAMPT-stimulated upregulation of MMP-1 and CCL2 (data not shown). Moreover, the upregulation of both molecules by NAMPT was also not affected by inhibitors against the p38, MEK1/2 and PI3K signaling pathways. However, SP600125, an inhibitor of JNK signaling, blocked completely the stimulatory effect of NAMPT on the MMP-1 expression at 1 d (Figure 2(b)). In addition, SP600125 reduced significantly the NAMPT-stimulated upregulation of CCL2 by 69% at this time point (Figure 2(b)). 

### 3.4. Stimulation of NAMPT Production by *P. gingivalis*

**Figure 3:**
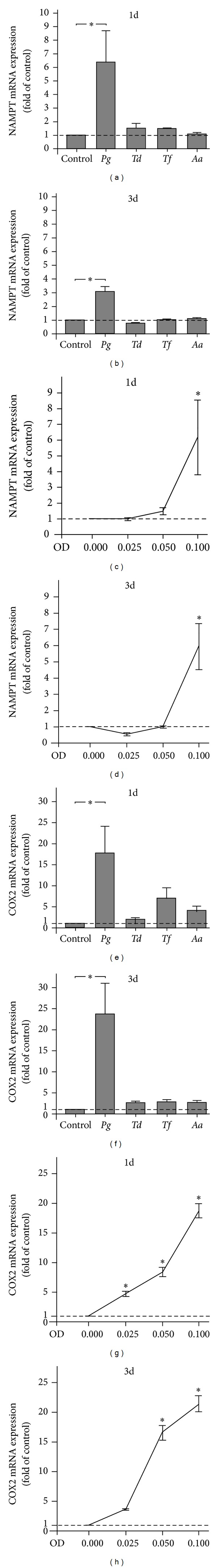
Regulation of NAMPT and COX2 expressions by periodontopathogens. NAMPT expression in PDL cells from 3 donors in response to various periodontopathogens (OD: 0.1; *P. gingivalis, Pg; T. denticola, Td; T. forsythia, Tf; A. actinomycetemcomitans, Aa) *at 1 d (a) and 3 d (b). Stimulation of NAMPT expression in PDL cells from 3 donors by various concentrations of *P. gingivalis* (OD: 0.025, 0.050, and 0.100) at 1 d (c) and 3 d (d). COX2 expression in PDL cells from 3 donors in response to various periodontopathogens (OD: 0.1; *P. gingivalis, Pg; T. denticola, Td; T. forsythia, Tf; A. actinomycetemcomitans, Aa*) at 1 d (e) and 3 d (f). Stimulation of COX2 expression in PDL cells from 3 donors by various concentrations of *P. gingivalis* (OD: 0.025, 0.050, and 0.100) at 1 d (g) and 3 d (h). All experiments were performed in triplicate and repeated at least twice. Mean ± SEM; *significantly (*P* < 0.05) different from *P. gingivalis*-untreated cells (control).

We then wondered whether pathogens that are strongly associated with periodontitis are capable of inducing the production of NAMPT in PDL cells. Whereas *T. denticola*,* T. forsythia *and *A. actinomycetemcomitans* had no significant effect, *P. gingivalis* increased significantly the NAMPT mRNA expression at 1 d and 3 d (Figures 3(a) and 3(b)). As shown in Figures 3(c) and 3(d), the actions of *P. gingivalis* on NAMPT mRNA expression were dose-dependent. Interestingly, pre-incubation with a specific inhibitor against NF*κ*B signaling blocked completely the *P. gingivalis*-induced stimulation of NAMPT at 1 d. Furthermore, inhibitors against JNK and p38 signaling reduced the stimulatory effects of NAMPT by 87% and 37%, respectively, at this time point. 

**Figure 4:**
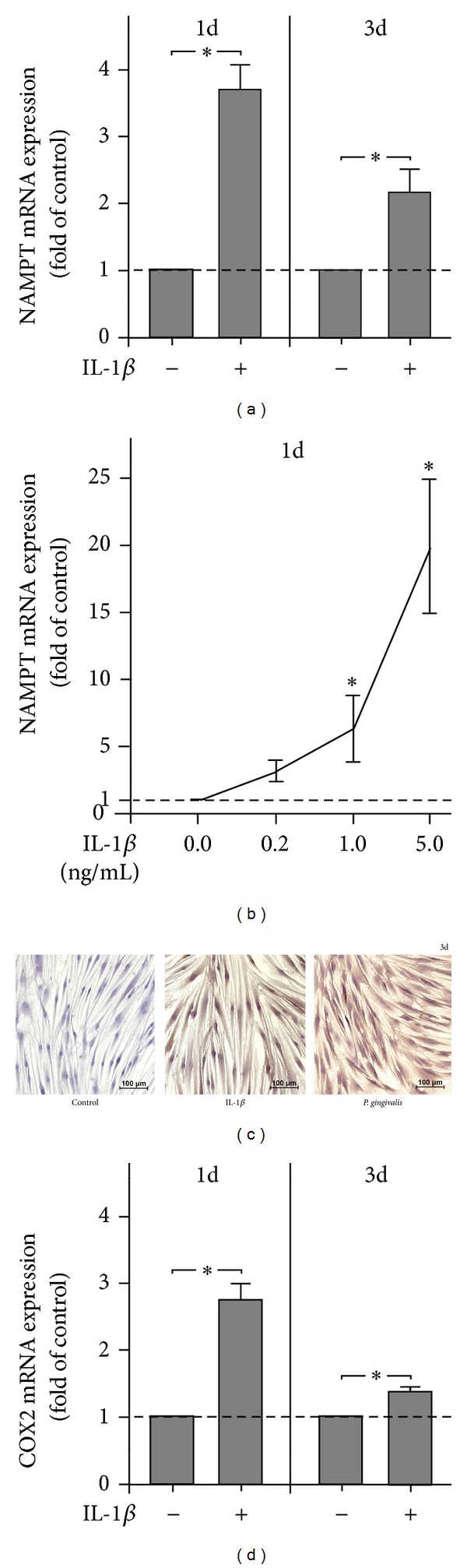
Regulation of NAMPT and COX2 expressions by IL-1*β*. NAMPT expression in PDL cells from 6 donors in response to IL-1*β* (1 ng/mL) at 1 d and 3 d (a). Stimulation of NAMPT expression in PDL cells from 3 donors by various concentrations of IL-1*β* (0.2, 1.0, and 5.0 ng/mL) at 1 d (b). NAMPT protein synthesis in PDL cells in the presence and absence of IL-1*β* and *P. gingivalis* at 3 d. Images from one representative donor are shown (c). COX2 expression in PDL cells from 6 donors in response to IL-1*β* (1 ng/mL) at 1 d and 3 d (d). All experiments were performed in triplicate and repeated at least twice. Mean ± SEM; *significantly (*P* < 0.05) different from IL-1*β*-untreated cells (control).

 The stimulation of NAMPT expression by *P. gingivalis* was also observed at protein level, as analyzed by ELISA: at 1 d, PDL cells in the presence and absence of *P. gingivalis* produced 28.83 ± 0.29 ng NAMPT protein/10^4^ cells and 17.91 ± 0.58 ng NAMPT protein/10^4^ cells, respectively. At 3 d, *P. gingivalis*-stimulated cells and control cells synthesized 30.97 ± 3.09 ng NAMPT protein/10^4^ cells and 21.65 ± 2.94 ng NAMPT protein/10^4^ cells, respectively. The differences between both groups at each time point were significant. The stimulation of NAMPT protein production by *P. gingivalis* was also evidenced by immunocytochemistry (Figure 4(c)).

 Like NAMPT, COX2 was significantly upregulated by *P. gingivalis* at 1 d and 3 d (Figures 3(e) and 3(f)). By contrast, all other periodontopathogens were not capable of stimulating the COX2 expression at both time points (Figures 3(e) and 3(f)). The *P. gingivalis*-induced stimulation of COX2 expression was dose-dependent (Figures 3(g) and 3(h)). 

### 3.5. Stimulation of NAMPT Production by IL-1*β*

Finally, we examined whether NAMPT is also induced by IL-1*β* in PDL cells. Interestingly, IL-1*β* upregulated significantly the NAMPT expression at 1 d and 3 d (Figure 4(a)). Moreover, the actions of IL-1*β* on NAMPT expression were dose-dependent at 1 d (Figure 1(b)) but not at 3 d (data not shown). When cells were preincubated with an inhibitor against the MEK1/2 signaling pathway, the IL-1*β*-induced stimulation of NAMPT expression was completely suppressed. Moreover, inhibitors against JNK, NF*κ*B, and p38 signaling reduced the stimulatory effect of IL-1*β* on NAMPT by 80%, 78%, and 46%, respectively. 

 The upregulation of NAMPT at transcriptional level was paralleled by increased NAMPT protein levels in the supernatants of IL-1*β*-stimulated cells: at 1 d, PDL cells in the presence and absence of IL-1*β* synthesized 32.68 ± 3.62 ng NAMPT protein/10^4^ cells and 20.28 ± 4.36 ng NAMPT protein/10^4^ cells, respectively. At 3 d, IL-1*β*-treated cells and control cells produced 30.97 ± 3.82 ng NAMPT protein/10^4^ cells and 20.43 ± 0.76 ng NAMPT protein/10^4^ cells, respectively. The differences between groups were significant at both time points. The stimulation of NAMPT protein synthesis by IL-1*β* was also observed by immunocytochemistry (Figure 4(c)). 

 IL-1*β* also induced a significant upregulation of COX2 expression at 1 d and 3 d (Figure 4(d)). 

## 4. Discussion

Our study shows for this first time that NAMPT stimulates the production of CCL2 and MMP-1 in human PDL cells, suggesting that NAMPT may contribute to periodontal inflammation and matrix destruction through the production of these molecules. Furthermore, NAMPT is induced by the periodontopathogen *P. gingivalis* and the proinflammatory cytokine IL-1*β* in PDL cells, which shows that microbial and inflammatory signals may use this adipokine for their detrimental effects on the periodontium.

 MMP-1 degrades specifically type I collagen and, additionally, types II, III, V, IX, and X collagen, thereby playing a critical role for modeling and remodeling of the extracellular matrix [25]. Several studies have revealed that MMP-1 levels are increased in GCF and human gingiva from periodontitis patients [26, 27]. Furthermore, MMP-1 levels could be significantly reduced by periodontal treatment [27]. CCL2 is synthesized by a variety of cell types and regulates the migration and infiltration of monocytes, memory T lymphocytes, and natural killer cells. In addition, CCL2 seem to influence T-cell immunity [28]. Higher levels of CCL2 in GCF and gingiva from inflamed sites have been reported [20–31]. Our experiments provide original evidence that NAMPT induces increased expression and synthesis of MMP-1 and CCL2 in human PDL cells, as evidenced by several assays and in a high number of donors. These findings are in accordance with previous observations in nonperiodontal cells and underline the proinflammatory and catabolic role of NAMPT in the pathophysiology of periodontitis [32–34]. Since increased NAMPT levels are found in obesity, our data suggest at least one mechanism whereby obesity could confer an increased risk of periodontitis in obese individuals [5, 9].

 Surprisingly, NAMPT also upregulated a number of genes, most of which have mainly been reported in oncology and neurosciences. The transcription factor EGR1 is a regulator of multiple tumor suppressors but, paradoxically, can also promote tumor progression [35, 36]. EGR1 is altered by hypoxia [37], stimulates angiogenesis [35], and regulates the immune system [38]. Synaptotagmins are transmembrane proteins with two Ca^2+^-binding C(2) domains in their cytosolic region. Syt7 regulates the exocytosis of lysosomes and thereby plays a role in bone resorption [39], cell migration [40], CTL responses [41], and neurodegeneration [42]. ITPKA phosphorylates inositol 1,4,5-trisphosphate, thereby modulating the calcium (Ca^2+^) level within the cell and the levels of a large number of inositol polyphosphates [43]. ITPKA promotes cell motility and the metastatic potential of tumor cells [43, 44]. Furthermore, ITPKA is critical for the spatial and temporal regulation of spine actin remodeling, synaptic plasticity, and learning and memory [45]. Little is known about neurotrimin, a neural cell adhesion protein. It has been shown to be an estrogen-regulated determinant of peripheral sympathetic innervation [46] and to play a role in axonal fasciculation of specific cerebellar systems and the formation of excitatory synapses and their stabilization [47]. The IGF2BPs bind to the 5′-untranslated region of IGF-2 mRNA and regulate a number of important aspects of cell function, such as cell polarization, morphology, migration, proliferation, differentiation, and invasion [48, 49]. NRP1 plays a critical part in neuronal development, in angiogenesis and tumor invasion [50, 51]. NRP1 is a coreceptor for members of the vascular endothelial growth factor family and the class 3 semaphorins, which are polypeptides with roles in axonal guidance [52]. Furthermore, NRP1 mediates interaction of regulatory T cells with dendritic cells and modulates the immune response [53]. KCTDs act as auxiliary subunits of GABA(B) receptors and generate desensitizing receptor responses [54]. Further studies should clarify the physiological and pathological role of these genes in the periodontium.

 We also sought to examine the intracellular mechanism whereby the stimulatory effects of NAMPT on MMP-1 and CCL2 are accomplished. Although NAMPT stimulated the NF*κ*B nuclear translocation in PDL cells, which concurs with observations in other cells [32, 55, 56], pre-incubation of cells with a specific NF*κ*B inhibitor did not abrogate the NAMPT actions on MMP-1 and CCL2 in our cells. However, the NF*κ*B signaling pathway could be involved in the NAMPT-induced upregulation of some of the other genes which were identified by microarray and PCR analyses. Interestingly, our experiments revealed that the NAMPT-induced upregulation of MMP-1 and CCL2 was JNK dependent, which is supported by findings from others [32].

 Recently, increased NAMPT levels have been found in GCF, gingival tissues, and serum from periodontally diseased patients, as compared to periodontally healthy individuals. This suggests that NAMPT might also be produced locally in the periodontium and regulated by periodontopathogens and/or inflammatory mediators [12, 13]. Interestingly, we found that *P. gingivalis*, a key pathogen associated with periodontitis, and the proinflammatory cytokine IL-1*β*, which is increased at inflamed periodontal sites, can induce NAMPT in PDL cells, thereby supporting the assumption that NAMPT is locally produced in the presence of an infectious and/or inflammatory environment. By upregulation of MMP-1 and CCL2, NAMPT may mediate and amplify proinflammatory and proteolytic actions of *P. gingivalis* and IL-1*β* on the PDL. The finding that NAMPT is induced by *P. gingivalis* is in line with our previous experiments, which have demonstrated that NAMPT is upregulated by *F. nucleatum*, a gram-negative, anaerobic microorganism, which acts as a bridge bacterium between early and late colonizers during plaque development [57]. Interestingly, *T. forsythia, T. denticola, and A. actinomycetemcomitans *did not regulate the NAMPT expression, at least at the concentration tested, in the present study. Our observation that NAMPT is increased by IL-1*β* in PDL cells concurs with findings in other cells, which have also been shown to produce increased NAMPT levels in response to IL-1*β* [58–60]. Our data provide evidence that PDL cells produce increased levels of NAMPT under infectious and inflammatory conditions, which suggests that local production of NAMPT in the inflamed periodontium could contribute to the increased gingival and serum levels of NAMPT, as observed in patients with periodontitis. NAMPT could therefore also represent a pathomechanistic link, how periodontitis affects systemic diseases, such as diabetes mellitus and cardiovascular diseases.

 Interestingly, the upregulation of NAMPT by *P. gingivalis* could be completely blocked by an inhibitor against NF*κ*B and reduced by inhibitors against the JNK and p38 signaling pathways. Recently, other investigators have also shown that *P. gingivalis* induces NF*κ*B activation and p38 signaling for its actions [61, 62]. An inhibitor against MEK1/2 signaling pathway suppressed completely the IL-1*β*-induced stimulation of NAMPT expression. JNK, NF*κ*B, and p38 signaling were also found to be involved in the stimulatory effects of IL-1*β* on NAMPT. That IL-1*β* exploits the NF*κ*B and mitogen-activated protein kinase pathways for its actions is well known and supported by the present findings [63, 64].

 In order to simulate an infectious environment, PDL cells were incubated with a suspension of various periodontopathogens. Since the suspensions were exposed to intensive ultrasonication, the suspensions contained disrupted cell wall particles with a high amount of lipopolysaccharide. However, other bacterial components may also have been present in the suspension. The concentrations used in this study were determined by dose-response experiments. However, a significant upregulation of NAMPT was only observed for *P. gingivalis*. *P. gingivalis* is a gram-negative bacterium and strongly associated with periodontitis. For its detrimental effects, *P. gingivalis* possesses a number of virulence factors, such as gingipains and fimbriae. *P. gingivalis* can invade host cells and also evade the host defense system [65–67]. As it has been shown in several cells that COX2, an enzyme which is responsible for the formation of prostanoids, is upregulated by *P. gingivalis* and IL-1*β*, we also analyzed the COX2 expression as a positive control in our study [61, 62, 68, 69]. As expected, *P. gingivalis* and IL-1*β* increased the COX2 expression in a dose- and time-dependent manner in PDL cells. 

 In summary, the present study demonstrates for the first time that NAMPT stimulates the production of MMP-1 and CCL2 in human PDL cells, which suggests that NAMPT may contribute to periodontal inflammation and matrix destruction through the production of these molecules. Therefore, increased NAMPT levels, as found in obesity, may represent at least one mechanism, whereby obesity could confer an increased risk of periodontitis in obese individuals. In addition, our study provides original evidence that NAMPT is induced by the periodontopathogen *P. gingivalis* and the proinflammatory cytokine IL-1*β* in PDL cells, which shows that microbial and inflammatory signals may use this adipokine for their detrimental effects on the periodontium. These findings suggest that local production of NAMPT in the inflamed PDL could contribute to the increased gingival and serum levels of NAMPT, as observed in patients with periodontitis and therefore represent a pathomechanistic link whereby periodontitis affects systemic diseases, such as diabetes mellitus and cardiovascular diseases.

## Figures and Tables

**Table 1:** Regulation of gene expression by NAMPT, as analyzed by microarray.

Gene	LogFC	adj. *P* value
EGR1	2.184	0.008
MMP-1	1.545	0.010
SYT7	1.443	0.010
ITPKA	1.292	0.010
CCL2	1.150	0.028
NTM	1.114	0.028
IGF2BP3	1.040	0.020
NRP1	1.024	0.010
KCTD12	1.019	0.048
HSPB3	−1.035	0.030
TM4SF20	−1.164	0.028
RGS4	−1.397	0.025

Effect of NAMPT on gene expression of PDL cells from 3 donors at 1 d. Only genes with statistically significant (FDR < 5%) logFC ≥ 1 (upregulation by at least 2-fold) or logFC ≤ −1 (downregulation by at least 50%) were considered. Abbreviations: EGR1: early growth response 1; MMP-1: matrix metalloproteinase-1; SYT7: synaptotagmin 7; ITPKA: inositol 1,4,5-trisphosphate 3-kinase a; CCL2: chemokine, cc motif, ligand 2; NTM: neurotrimin; IGF2BP3: insulin-like growth factor 2 mRNA-binding protein 3; NRP1: neuropilin 1; KCTD12: potassium channel tetramerization domain-containing protein 12; HSPB3: heat-shock 27-kD protein 3; TM4SF20: transmembrane 4 L six family member 20; RGS4: regulator of G protein signaling 4.

**Table 2:** Upregulation of gene expression by NAMPT, as analyzed by real-time PCR.

Gene	Fold change (mean)	SD	SEM	*P* value
EGR1	2.422	2.306	0.421	<0.001
MMP-1	3.781	3.116	0.569	<0.001
SYT7	4.575	7.947	1.451	<0.001
ITPKA	2.764	3.182	0.581	0.001
CCL2	2.285	1.346	0.246	<0.001
NTM	2.124	2.460	0.449	<0.001
IGF2BP3	1.897	0.970	0.177	<0.001
NRP1	1.653	1.658	0.303	<0.001
KCTD12	1.713	0.958	0.175	0.095

Effect of NAMPT on gene expression of PDL cells from 10 donors at 1 d. Abbreviations: EGR1: early growth response 1; MMP-1: matrix metalloproteinase-1; SYT7: synaptotagmin 7; ITPKA: inositol 1,4,5-trisphosphate 3-kinase a; CCL2: chemokine, cc motif, ligand 2; NTM: neurotrimin; IGF2BP3: insulin-like growth factor 2 mRNA-binding protein 3; NRP1: neuropilin 1; KCTD12: potassium channel tetramerization domain-containing protein 12.
